# Living donor kidney transplantation in a patient with inherited skin fragility disorder in a resource-limited setting: a case report

**DOI:** 10.1093/jscr/rjaf984

**Published:** 2025-12-13

**Authors:** Sasho Dohchev, Aleksandar Trifunovski, Dimitar Trajkovski, Josif Janchulev, Aleksandra Gavrilovska Brzanov, Zaklina Shterjova Markovska, Ivana Dohcheva Karajovanov, Irena Rambabova Bushljetik, Goce Spasovski

**Affiliations:** University Clinic for Urology Faculty of Medicine, Ss. Cyril and Methodius University, Majka Tereza 17, 1000 Skopje, RN Macedonia; University Clinic for Urology Faculty of Medicine, Ss. Cyril and Methodius University, Majka Tereza 17, 1000 Skopje, RN Macedonia; University Clinic for Urology Faculty of Medicine, Ss. Cyril and Methodius University, Majka Tereza 17, 1000 Skopje, RN Macedonia; University Clinic for Urology Faculty of Medicine, Ss. Cyril and Methodius University, Majka Tereza 17, 1000 Skopje, RN Macedonia; Department for Anesthesia, Reanimation, and Intensive Care, University Clinic for Traumatology Orthopedic Disease, Faculty of Medicine, Ss. Cyril and Methodius University, Majka Tereza 17, 1000 Skopje, RN Macedonia; University Clinic for Nephrology Faculty of Medicine, Ss. Cyril and Methodius University, Majka Tereza 17, 1000 Skopje, RN Macedonia; University Clinic for Dermatology Faculty of Medicine, Ss. Cyril and Methodius University, Majka Tereza 17, 1000 Skopje, RN Macedonia; University Clinic for Nephrology Faculty of Medicine, Ss. Cyril and Methodius University, Majka Tereza 17, 1000 Skopje, RN Macedonia; University Clinic for Nephrology Faculty of Medicine, Ss. Cyril and Methodius University, Majka Tereza 17, 1000 Skopje, RN Macedonia

**Keywords:** end-stage renal disease, epidermolysis bullosa dystrophica, inherited skin fragility disorder, kidney transplantation

## Abstract

Kidney transplantation in individuals with congenital skin fragility diseases is exceedingly uncommon due to perioperative concerns such as compromised wound healing, respiratory complications, and issues with vascular access. Herein, we report a case of successful living-donor kidney transplantation in a 37-year-old male with dystrophic epidermolysis bullosa and end-stage renal disease. Multidisciplinary planning, customized anesthesia, and protective intraoperative measures are essential to avoid these problems. Epidural anesthesia combined with mild sedation facilitated surgery without the need for airway instrumentation, while meticulous handling maintained skin integrity. The operation and recovery were unremarkable, and the graft function was maintained at 12 months. This case demonstrates that complex transplantation is achievable in environments with limited resources, through personalized perioperative treatment and interdisciplinary cooperation.

## Introduction

Kidney transplantation (KT) remains the optimal treatment for end-stage renal disease (ESRD), enhancing both quality of life and survival rates. Epidermolysis bullosa (EB) and kindred hereditary skin fragility disorders provide specific perioperative challenges that necessitate interdisciplinary management. Given the organ scarcity, living donor kidney transplantation is imperative [1].

Over 30 subtypes of EB (simplex, junctional, dystrophic, and Kindler) are characterized by blistering, chronic wounds, scarring, contractures, infections, and skin/mucosal fragility; severe manifestations sometimes lead to premature mortality. Renal illness is common, especially in patients with recessive dystrophic EB [2–4].

Surgery entails risks due to comorbidities, delicate skin, and complicated airways. Despite the limited global incidence of KT cases, meticulous preoperative assessment, wound and airway safeguarding, and tailored immunosuppressive are required [3, 5–7].

We illustrate the significance of multidisciplinary planning and meticulous perioperative management by presenting a living donor kidney transplantation in a patient with end-stage renal disease and a unique genodermatosis.

## Case presentation

A 37-year-old male with a lifelong history of skin fragility, blisters, and joint contractures was clinically diagnosed with inherited epidermolysis bullosa dystrophica at birth, supported by a positive family history (father with identical manifestations; genetic testing was initially deferred). Over the past few decades, he developed dystrophic nails, limb mutilations, and recurrent skin infections.

**Figure 1:**
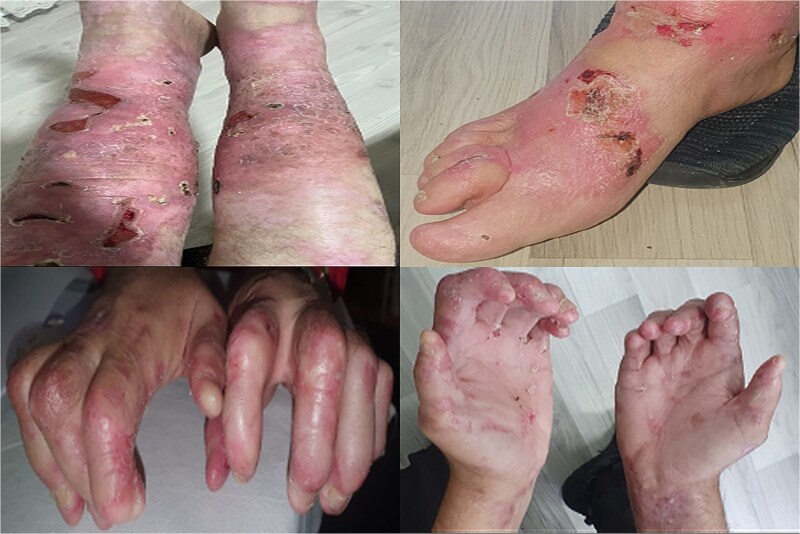
Ruptured sero-hemorrhagic blisters, eroded areas, inflamed, erythematous, fragile skin, some scarring mainly localized on upper and lower extremities, dystrophic nails, and contractures of hands and feet.

In 2019, crescentic glomerulonephritis with stage 3 CKD was diagnosed due to elevated serum creatinine levels and proteinuria. By 2022, the illness had advanced to ESRD and required hemodialysis despite the use of corticosteroids and cyclophosphamide. An interdisciplinary team, including nephrologists, dermatologists, anesthesiologists, and surgeons, evaluated the feasibility of KT. The patient exhibited widespread erosion and serohemorrhagic blisters involving the extremities, trunk, and ears, along with hypertrophic dystrophic nails (Fig. 1).

**Figure 2:**
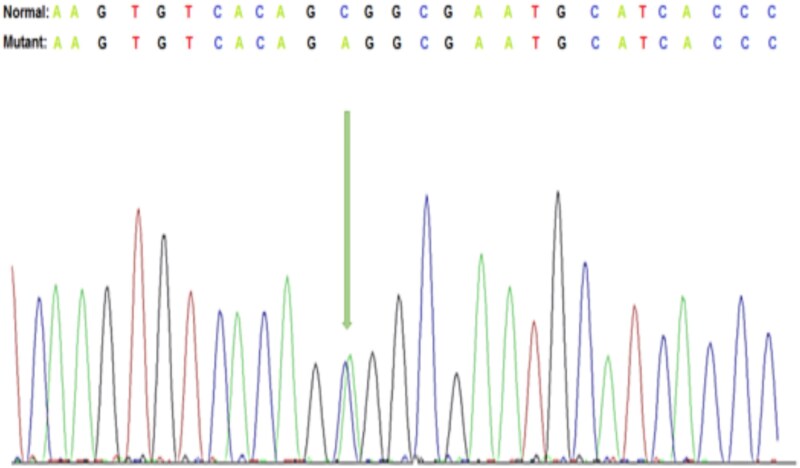
Representative Sanger DNA sequencing results showing the detection of c.373 C > A, p.(Pro125Thr) in exon 1 of the *KRT17* gene.

Targeted massively parallel sequencing genetic testing revealed a heterozygous variant of KRT17 (c.373C > A; p.Pro125Thr, exon 1) of unknown significance (Fig. 2). Given the father's history of similar skin manifestations, this variant was assumed to be inherited from the father, as it was absent from the mother. Autosomal dominant pachyonychia congenita (PC) has been linked to pathogenic variants of KRT17. The patient had cutaneous fragility and nail dystrophy that partially overlapped with PC.

The patient’s sister, with haploidentical HLA compatibility and a negative cross-match, served as a living donor. In September 2022, she underwent hand-assisted laparoscopic left nephrectomy, yielding a graft with three arteries, one vein, and a ureter, with a minimal warm ischemia time (WIT) of 5 s and 15 s. Following completion of the necessary hemostasis procedures in the donor, the kidney was drained from the preservation fluid.

### Anesthesia and surgery management

Standard monitoring with electrocardiography, peripheral pulse oximetry, and invasive blood pressure measurements was initiated, and handling strategies were adapted to minimize friction and pressure on the skin. Non-adhesive materials and silicone-based padding were applied to all the contact points. Arterial cannulation for invasive blood pressure monitoring was performed under local anesthesia and ultrasound guidance, with utmost care to avoid skin trauma. Central venous catheterization was performed under ultrasound guidance as part of the standard protocol for central venous pressure monitoring and fluid therapy. Regional anesthesia was provided via an epidural catheter placed at the Th11–Th12 interspace under strict aseptic conditions. Following successful catheterization, sensory and motor blockade were achieved using 15 ml of 0.25% bupivacaine. The sedation was maintained with a continuous intravenous infusion of dexmedetomidine at 0.5 mcg/kg/min, ensuring the patient remained conscious, arousable, and cooperative throughout the procedure itself. The combination of neuraxial blockade and light sedation allows excellent intraoperative conditions while circumventing airway manipulation. The hemodynamic stability was maintained without the need for additional analgesics or sedatives. The patient remained haemodynamically stable throughout the surgery. Specific non-adhesive protective dressings were used to secure the arterial line and venous and epidural catheters to prevent blister formation and to maintain skin integrity.

**Figure 3:**
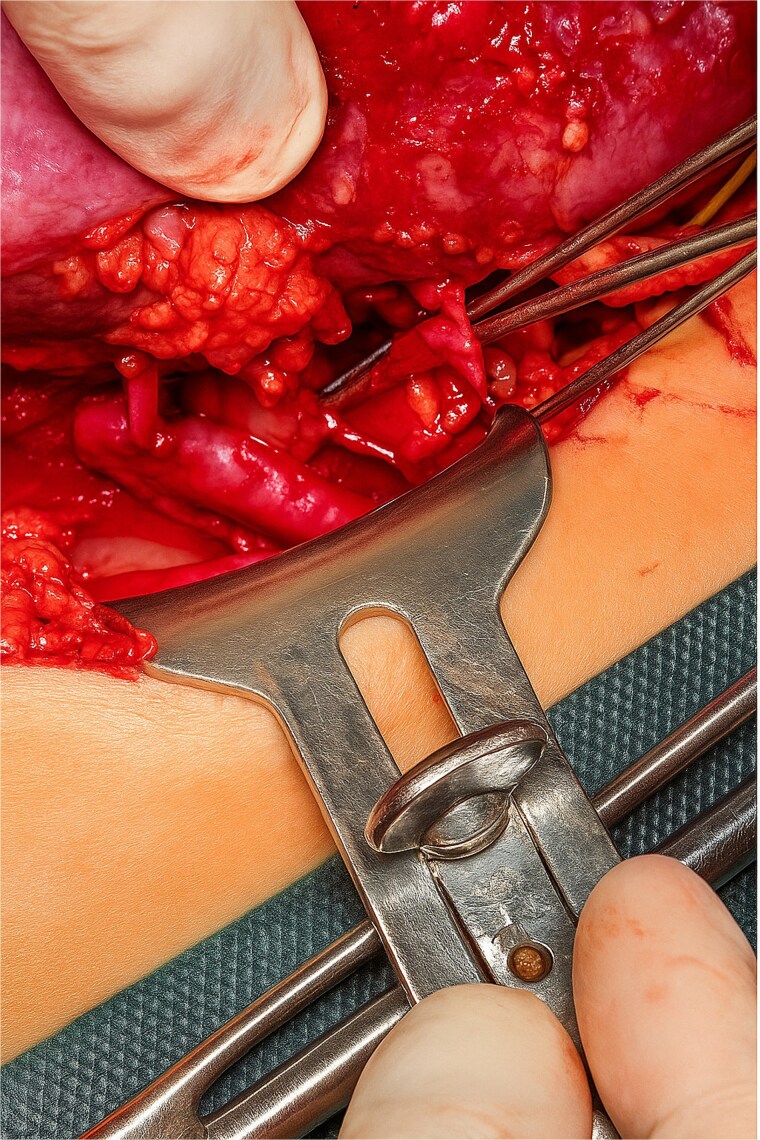
Anastomoses between the donor renal and recipient artery.

A Gibson incision was made, and dissection of the layers continued until the retroperitoneal space in the iliac fossa was reached. The iliac vessels were exposed and any identified lymphatic vessels were ligated. Following heparin administration at a dose of 100 U/kg, the external iliac vessels were clamped to allow for vascular anastomosis. Terminolateral anastomoses were performed between the donor renal vein and the recipient external iliac vein and between the two donor renal arteries and the recipient external iliac artery. Another terminolateral anastomosis was performed between the donor’s third renal artery and recipient’s inferior epigastric artery (Fig. 3). The kidneys were then perfused again. The first drop in urine was observed 40 s after reperfusion, indicating immediate graft function, and the ureter was anastomosed to the bladder via ureteroneocystostomy by using the lich-Gregoir technique. Hemostasis was achieved, and the wound was closed in layers. The procedure was completed without any complications.

**Figure 4:**
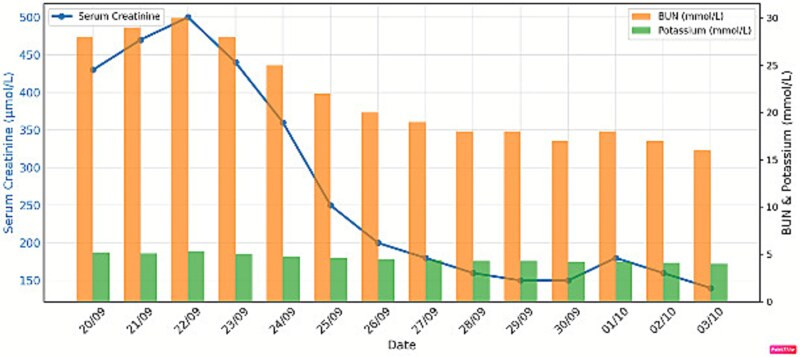
Renal function before and after living donor kidney transplant.

Post-transplant immunosuppression consisted of cyclosporine, corticosteroids, mycophenolic acid, and intravenous immunoglobulins along with antibiotic prophylaxis and supportive care. The graft functioned immediately, with a creatinine level of 150 μmol/L at discharge after 12 days (Fig. 4). No blistering, rejection, or major complications were observed during the first 12 months of follow-up.

## Discussion

This case underscores the amalgamation of genetic, clinical, anesthetic, surgical, and postoperative elements in the management of a patient with hereditary skin fragility undergoing living-donor KT.

A heterozygous KRT17 mutation (c.373C > A; p.Pro125Thr) confounded the diagnosis, exhibiting overlap with pachyonychia congenita but devoid of mucosal manifestations, aligning with broader keratin-associated symptoms [6, 7]. In accordance with American College of Medical Genetics and Genomics (ACMG) standards, care prioritized clinical phenotype over variants of uncertain significance [8, 9].

Haploidentical kidney transplantation, infrequently utilized in uncommon genetic disorders, was achievable with a negative cross-match, corroborating previous findings. Notwithstanding the presence of several renal arteries, the vascular anastomoses were accomplished successfully [10]. Epidural anesthesia alone, unique in EB/EB-like KT, mitigated intubation hazards, maintained spontaneous respiration, and reduced trauma, in contrast to previous general or mixed anesthesia methods [3, 5, 11].

Immunosuppression with intravenous immunoglobulin (IVIG), corticosteroids, mycophenolic acid, and cyclosporine was chosen; cyclosporine inhibited Th17, alleviated EB symptoms, and preserved graft stability compared to tacrolimus [5, 6, 12]. Graft function was maintained for almost 12 months without rejection or blistering. This example highlights the viability of haploidentical kidney transplantation with customized anesthetic and immunosuppressive, even in resource-constrained environments, underscoring the importance of multidisciplinary collaboration.
